# Identification of an *ADAMTS2* frameshift variant in a cat family with Ehlers–Danlos syndrome

**DOI:** 10.1093/g3journal/jkad152

**Published:** 2023-07-18

**Authors:** Rebecca Simon, Sarah Kiener, Nina Thom, Laura Schäfer, Janina Müller, Elfi K Schlohsarczyk, Ulrich Gärtner, Christiane Herden, Tosso Leeb, Gesine Lühken

**Affiliations:** Institute of Animal Breeding and Genetics, Justus Liebig University, Giessen 35390, Germany; Institute of Genetics, Vetsuisse Faculty, University of Bern, Bern 3001, Switzerland; Dermfocus, University of Bern, Bern 3001, Switzerland; Small Animal Clinic, Justus Liebig University, Giessen 35392, Germany; Small Animal Clinic, Justus Liebig University, Giessen 35392, Germany; Institute of Veterinary Pathology, Justus Liebig University, Giessen 35392, Germany; Institute of Veterinary Pathology, Justus Liebig University, Giessen 35392, Germany; Institute of Anatomy and Cell Biology, Justus Liebig University, Giessen 35385, Germany; Institute of Veterinary Pathology, Justus Liebig University, Giessen 35392, Germany; Institute of Genetics, Vetsuisse Faculty, University of Bern, Bern 3001, Switzerland; Dermfocus, University of Bern, Bern 3001, Switzerland; Institute of Animal Breeding and Genetics, Justus Liebig University, Giessen 35390, Germany

**Keywords:** skin, dermatology, dermatosparaxis, veterinary medicine, *Felis catus*, whole genome sequence, precision medicine

## Abstract

We investigated 4 European domestic shorthair kittens with skin lesions consistent with the dermatosparaxis type of the Ehlers–Danlos syndrome, a connective tissue disorder. The kittens were sired by the same tomcat but were born by 3 different mothers. The kittens had easily torn skin resulting in nonhealing skin wounds. Both clinically and histologically, the skin showed thin epidermis in addition to inflammatory changes. Changes in collagen fibers were visible in electron micrographs. The complete genome of an affected kitten was sequenced. A one base pair duplication leading to a frameshift in the candidate gene *ADAMTS2* was identified, p.(Ser235fs*3). All 4 affected cats carried the frameshift duplication in a homozygous state. Genotypes at this variant showed perfect cosegregation with the autosomal recessive Ehlers–Danlos syndrome phenotype in the available family. The mutant allele did not occur in 48 unrelated control cats. *ADAMTS2* loss-of-function variants cause autosomal recessive forms of Ehlers–Danlos syndrome in humans, mice, dogs, cattle, and sheep. The available evidence from our investigation together with the functional knowledge on *ADAMTS2* in other species allows to classify the identified *ADAMTS2* variant as pathogenic and most likely causative variant for the observed Ehlers–Danlos syndrome.

## Introduction

The Ehlers–Danlos syndrome (EDS, *Fibrodysplasia elastica*) represents a group of hereditary disorders associated with defects in collagen biosynthesis (Malfait *et al*. 2017). This heterogenous group of connective tissue disorders is named after Edvard Ehlers and Henri-Alexandre Danlos who independently described human patients with the syndrome in detail at the beginning of the 19th century (Parapia and Jackson 2008).

Signs may vary between species and the different types of EDS, e.g. joint hypermobility is primarily observed in humans. However, a universally occurring feature is the hyperelasticity of the skin and the resulting tendency to skin tears. One type of EDS, called dermatosparaxis EDS (dEDS), was first observed, and its biochemical background was described in detail in cattle (Lapière *et al*. 1971) (OMIA 000328-9913). The connective tissue shows alterations of its normal structure due to a deficiency of the enzyme procollagen peptidase, which catalyzes the formation of type 1 procollagen (Lapière and Nusgens 1993). The structurally abnormal dermal collagen shows decreased strength; therefore, skin wounds can already be caused by minimal trauma like handling or even normal activity (Counts *et al*. 1980; Crosaz *et al*. 2013; Hargis and Myers 2017). Histologically, dermatosparaxis skin shows variations in terms of the caliber of collagen fibers, which are irregular, undirected, and loosely arranged (Colige *et al*. 2004; van Damme *et al*. 2016). Variants in the *ADAMTS2* gene are known to cause dermatosparaxis in humans (Colige *et al*. 2004; van Damme *et al*. 2016) as well as in cattle (Colige *et al*. 1999), sheep (Zhou *et al*. 2012; Monteagudo *et al*. 2015; Joller *et al*. 2017), and dogs (Jaffey *et al*. 2019). The *ADAMTS2* gene encodes ADAM metallopeptidase with thrombospondin type 1 motif 2, also termed procollagen I N-proteinase, which cleaves the propeptides of procollagen type I and II (Wang *et al*. 2003). The role of different ADAMTS proteases for normal collagen biosynthesis and in dEDS has been reported (Le Goff *et al*. 2006).

Different forms of EDS are known to occur in humans (Malfait *et al*. 2017, 2020) as well as in several animal species, including cattle (Hanset and Lapiere 1974; Carty *et al*. 2016; Jacinto *et al*. 2020), dog (Bauer *et al*. 2019; Jaffey *et al*. 2019; Kiener *et al*. 2022b), sheep (Joller *et al*. 2017), cat (Counts *et al*. 1980; Weingart *et al*. 2014; Spycher *et al*. 2018), horse (Eßer *et al*. 1999), and mink (Hegreberg 1975). Diagnosis is mainly based on the clinical appearance of the affected animal, histopathological examination of the collagen fibrils, and genetic analyses. In domestic cats, until now, only one gene, *COL5A1*, was reported to be involved in autosomal-dominant EDS (Spycher *et al*. 2018; Kiener *et al*. 2022a). Herein. we report the results of a comprehensive clinical, pathological and genetic analysis of dEDS in a cat family.

## Materials and methods

### Ethics statement

All cats in this study were privately owned. The index case, a deceased kitten, was transferred to the Institute of Veterinary Pathology of the Justus Liebig University Giessen for diagnostic purposes. The other 3 affected kittens were examined and treated at the Small Animal Clinic of the Justus Liebig University Giessen. All animals in this study were examined with the consent of the owner and handled according to good ethical standards. The “Cantonal Committee for Animal Experiments” (Canton of Bern; permit 71/19) and the Regional Council of Gießen (reference number 19 c 20 15 h 02 Gi 19/1 KTV 22/2020) approved the collection of samples from control cats.

### Animals

A group of free roaming farm cats (European domestic shorthair) is presented here. Initially, one female kitten with skin lesions resembling the appearance of dermatosparaxis was found dead. Later, 3 additional affected kittens were observed in 2 subsequent litters. All 3 litters that produced affected kittens were apparently from the same sire (tomcat). Subsequently, as many cats as possible (*n* = 27) from this semi-feral population, including mothers, littermates, and the tomcat, were captured for sampling and visual inspection. Despite the free roaming lifestyle of the cats, the farmer and owner of the cats was able to provide information about the kinship of the population.

### Clinical and pathological examination

Standard clinical and pathological examinations were done. Necropsy was performed on all affected kittens, and representative organ samples were fixed in 10% neutral buffered formalin, embedded in paraffin, and stained with hematoxylin and eosin (HE). Additionally, histochemical stains were performed on the skin, included periodic acid–Schiff reaction (PAS) and Masson trichrome stain. The skin of one affected kitten was examined by transmission electron microscopy. For this purpose, skin samples were fixed with 1.5% glutaraldehyde and 1.5% formaldehyde (freshly made from paraformaldehyde) in 0.15 M HEPES buffer. For epoxy resin embedding, cells were postfixed in 1% osmium tetroxide in aqua bidest, stained in half-saturated watery uranyl acetate, dehydrated in an ascending ethanol series, and finally embedded in Agar 100 (Agar scientific Ltd., UK). Ultrathin sections were cut using an ultramicrotome (Reichert Ultracut E, Leica) and examined with a transmission electron microscope (Zeiss EM 902). Digital images were captured with a slow-scan 2 K CCD camera (TRS, Tröndle, Moorenweis, Germany).

### DNA extraction

For the purpose of whole genome sequencing, genomic DNA was isolated from muscle tissue of the deceased kitten using a Maxwell RSC Tissue DNA Kit and a Maxwell RSC instrument (Promega, Dübendorf, Switzerland). For genotyping, DNA extraction from buccal swaps (sterile transport swabs; COPAN Italia SpA, Brescia, Italy) was executed using the Gentra Puregene Tissue Kit (QIAGEN GmbH, Hilden, Germany) following the manufacturer's instructions.

### Whole genome sequencing, variant calling, and variant filtering

An Illumina TruSeq PCR-free DNA library with ∼330 bp insert size of the deceased affected cat was prepared and sequenced on a NovaSeq 6000 instrument with 23× coverage (Illumina, San Diego, CA, USA). The sequence data were submitted to the European Nucleotide Archive with the study accession PRJEB7401 and sample accession SAMEA7376282. Mapping and alignment to the F.catus Fca126 mat1.0 reference genome assembly were performed as described (Jagannathan *et al*. 2019). Variant calling was performed using GATK HaplotypeCaller (McKenna *et al*. 2010) in gVCF mode as described (Jagannathan *et al*. 2019). Functional effects of the called variants were predicted with the SnpEff version 4.3t software (Cingolani *et al*. 2012) together with NCBI annotation release 105 for the F.catus_Fca126_mat1.0 genome reference assembly.

For variant filtering, we used 77 control genomes (Supplementary Table 1). A hard filtering strategy was employed, which required either a homozygous alternate (1/1) or heterozygous (0/1) genotype call in the affected kitten, while the 77 control cats were required to have either a homozygous reference (0/0) or missing (./.) genotype call in the vcf-file (Supplementary Table 2). Variants in 20 known functional candidate genes for EDS obtained from Kiener *et al*. 2022b were prioritized.

### Genotyping by Sanger sequencing

The *ADAMTS2* variant was genotyped by Sanger sequencing of PCR amplicons [XM_023254116.2:c.698dup or ChrA1:90,995,621dup (F.catus_Fca126_mat1.0 assembly)]. A forward primer 5′-TTCAATGTACCTGGCAAGCC-3′ and a reverse primer 5′-ATGCTGCAGATGGTGACTAC-3′ were designed with the software Primer3 (Untergasser *et al*. 2012) to produce a fragment with a size of 169 bp (wild type) or 170 bp (mutant) with standard PCR conditions. Purified PCR products were sent to LGC Genomics GmbH, Berlin (Germany), for Sanger sequencing, using the reverse primer. A similar approach was used to genotype the *COL1A2*:XM_003982764.6:c.2384G > A variant, using 5′- TCCCTAGAGCTGCCATTGAT-3′ and 5′- GAGGCAAGGTTGTTTGGCTA-3′ as forward and reverse primers, respectively (152 bp fragment size).

### Parentage testing

A DNA profile, based on 16 microsatellite markers, for parentage verification was commissioned from Laboklin GmbH & Co KG (Bad Kissingen, Germany). It was carried out with genomic DNA from the 3 mother cats, the 4 affected kittens, and the presumed father.

## Results

### Clinical and pathological findings

**Fig. 1.**
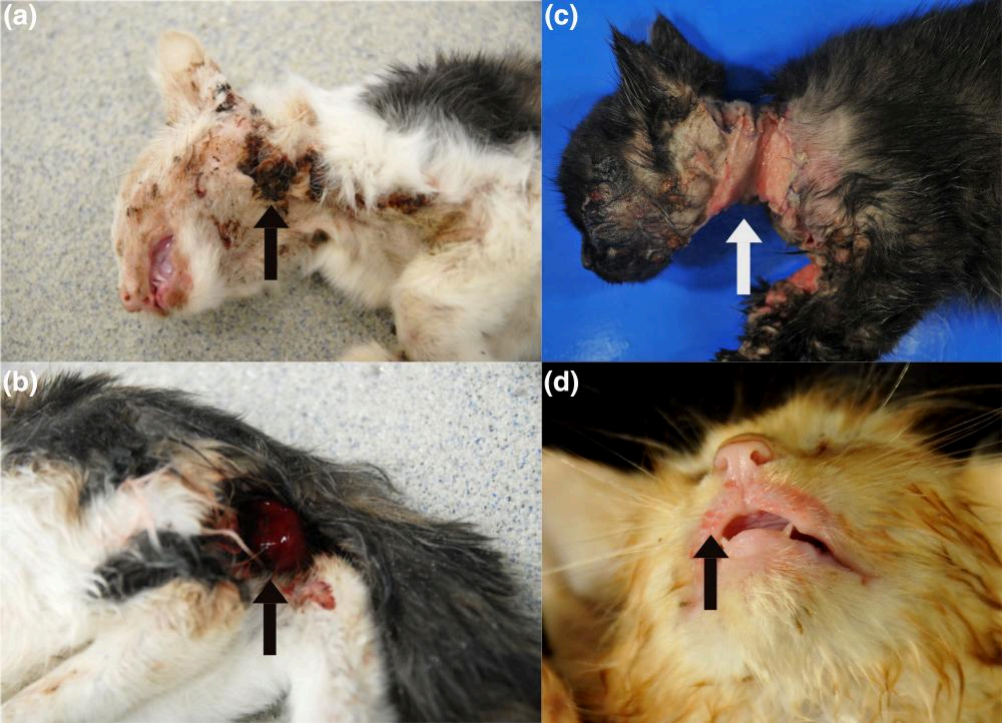
Gross condition of the deceased affected kittens (a and b initial case; c and d from second and third litter). a) Almost the entire head and the left neck showed extensive alopecia and severe multifocal ulcerative and purulent dermatitis occasionally accompanied by barky, dark-brown crust formation (arrow). The oral mucosa was moderately anemic. b) A rectal prolapse was also present in the initial case (arrow). c) After surgical treatment: severe loss of the epidermis especially in the cranial body regions (head, neck, forelimbs) with severe ulcerative partly crustose dermatitis and fragile skin, that tore at light touch (arrow); d) Kitten with a milder course, gross lesions were found exclusively on the head (temples and mucocutaneous junctions) with mild to moderate ulceration and crusting (arrow).

**Fig. 2.**
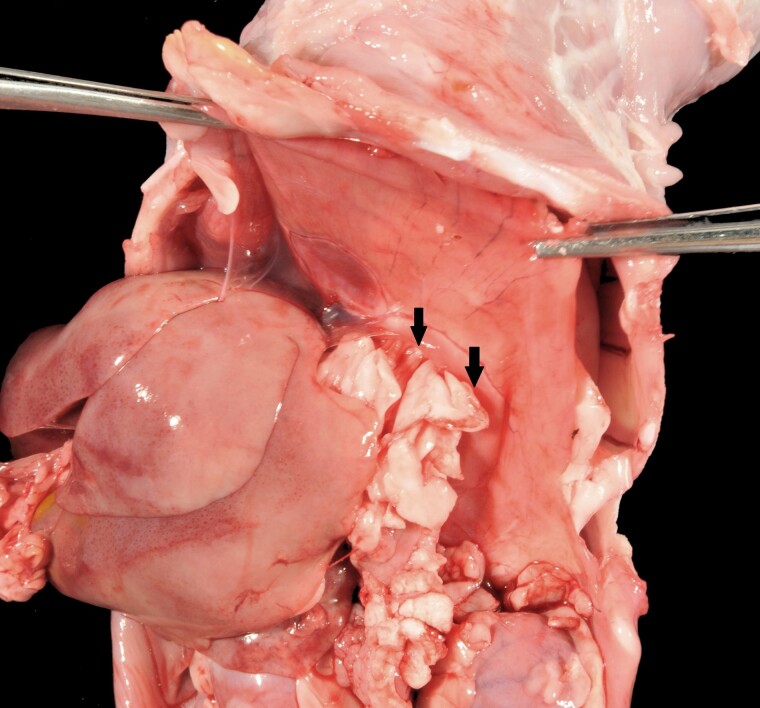
Abdominal cavity (lower and middle part of the figure) with a diaphragmatic hernia (arrows). The stomach and large parts of the omentum majus passed through this defect into the thoracic cavity, which is located behind the diaphragm in the upper part of the figure.

The initial case, a deceased female kitten of unknown age was in good body condition (weight: 1 kg). Body and tissues were affected by moderate postmortem changes. In addition to moderate anemia, the skin was markedly thin and soft and was easily torn. Large portions of the head, as well as the left side of the neck, exhibited extensive alopecia and severe multifocal ulcerative and purulent dermatitis, occasionally accompanied by partially detachable dark brown crusts up to 1 cm thick (Fig. 1a). Additionally, there was a prolapse of the rectum (Fig. 1b) as well as an invagination in the colon involving 3 cm of the large intestine with venous infarction of the invaginated part (intussusceptum). A diaphragmatic hernia, through which the stomach and large portions of the omentum majus entered the thoracic cavity (Fig. 2), was also observed. The urea concentration in the aqueous humor was 20 mmol/L (reference value: 5.0–11.3 mmol/L).

Three additional affected kittens from the following litters showed similar dermatological lesions as the first kitten (Fig. 1c and d). During handling, the skin was easily torn, and preexisting wounds were exacerbated even by gentle manipulation. Wounds in different stages and sizes were present. The head, ventral neck and front legs, and axillar region were most severely affected in all 3 cats; distribution of the lesions was more or less symmetrical. In addition, these kittens showed significantly reduced growth compared to their unaffected littermates. One of the cats was euthanized at first presentation due to an impaired general condition. In 2 of the 3 kittens, a symptomatic therapy with topical wound care and systemic anti-infective treatment were attempted, but due to progressive deterioration, both cats were humanely euthanized 5 days and 33 days after start of therapy, respectively.

**Fig. 3.**
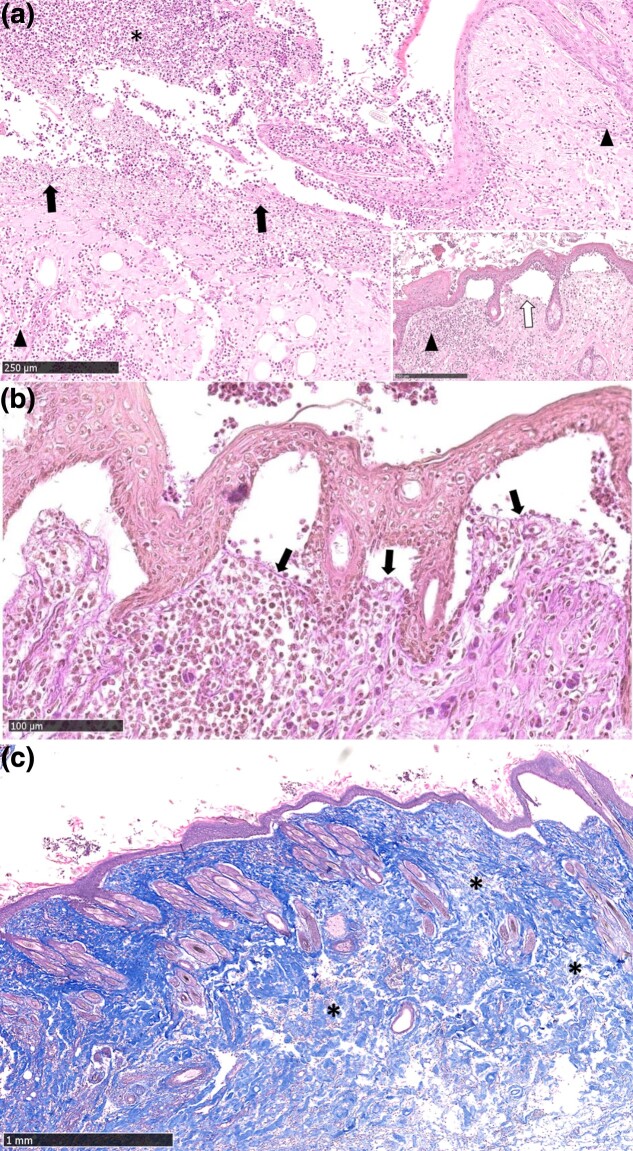
Skin of the head of an affected kitten. a) HE stain: severe chronic pyogranulomatous (arrowhead) and severe acute ulcerative and suppurative dermatitis (solid arrows) accompanied by serocellular crusts which contained bacteria (asterisk). Cleft-formation (open arrows) was observed at the dermo-epithelial junction adjacent to the ulcerative lesions. b) PAS reaction: cleft-formation at the dermo-epithelial junction. The basement membrane zone (arrows) formed the floor of the cleft. c) Masson trichrome stain: collagen fibers were stained uniformly blue and loosely arranged (asterisks) in the area of the clefts.

Histological examination revealed that the skin of the initial case was multifocally affected by both a mild to severe chronic pyogranulomatous and an acute ulcerative and suppurative dermatitis accompanied by serocellular crust formation, which contained bacteria (Fig. 3a). Adjacent to the ulcerative lesions, cleft-formation at the dermo-epithelial junction was often observed (Fig. 3a). The PAS reaction revealed that the basement membrane zone formed the floor of the cleft (Fig. 3b). The collagen fibers stained uniformly blue with Masson Trichrome stain (Fig. 3c) and showed a loose arrangement around the clefts. In the unaffected skin epidermis, dermis and adnexa were present and the collagen fibers were arranged physiologically. The invagination in the colon was accompanied by a moderate to severe chronic suppurative colitis characterized by a moderate to high amount of mononuclear cells infiltrating the intussusceptum, while the part of the colon containing the invaginated part (intussuscipiens) was infiltrated with macrophages and neutrophil granulocytes.

**Fig. 4.**
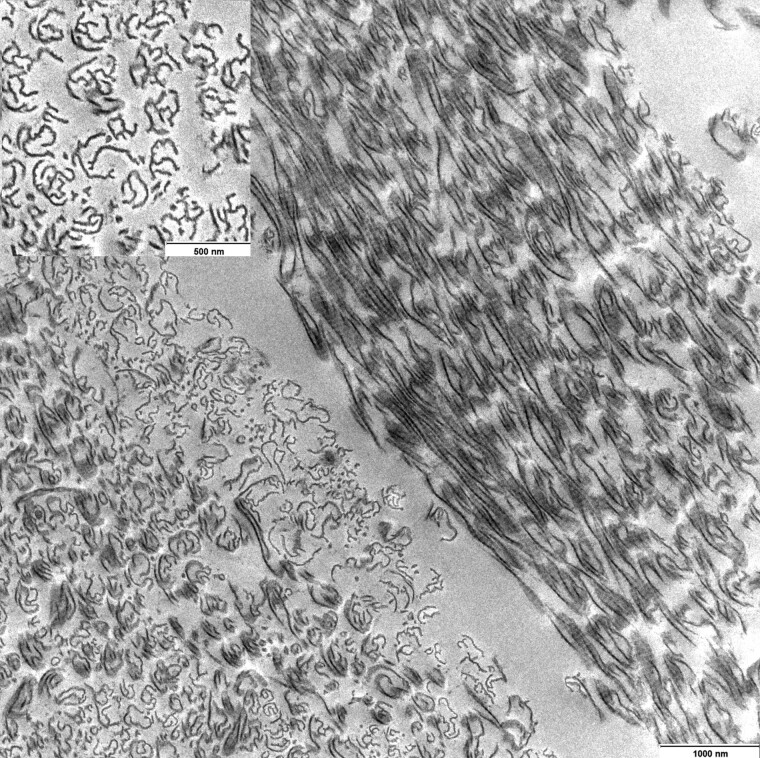
Longitudinal and cross section of collagen fibers (affected kitten): thin ribbon-like electron-dense fibrils appear disordered with an electron-lucent central area (hollow appearance).

Electron microscopy of the skin of one of the affected kittens showed severe abnormalities in the collagen fibers. The longitudinal section showed electron-loose parts framed by electron-dense filaments suggesting an “empty-tube” appearance. Cross section of collagen fibers showed electron dense ribbon-like structures up to 250 nm in diameter (Fig. 4).

### Genetic analyses

The genome of one affected cat was sequenced at 23× coverage. Genome sequences from 77 cats representing 14 breeds and 35 random-bred individuals and one of unknown origin were used as controls. Filtering for private protein-changing variants in the affected cat identified 2 potentially pathogenic variants in known EDS candidate genes, a heterozygous missense variant in *COL1A2*, and a homozygous frameshift variant in the *ADAMTS2* gene (Table 1; Supplementary Table 2). Genotyping of cats from the pedigree excluded the *COL5A1* variant as the genotypes did not cosegregate with the EDS phenotype, and 4 unaffected cats were homozygous for the mutant allele (Supplementary Table 3). The *COL1A2* variant was XM_003982764.5:c.2384G > A or XP_003982813.1:p.(Arg795Gln).

**Table 1.** Results of variant filtering in the affected cat against 77 control genomes.

Filtering step	Heterozygous	Homozygous
All variants in the sequenced cat	6,011,674	5,983,799
Private variants	70,995	20,434
Protein-changing private variants	353	81
Protein-changing private variants in functional candidate genes	1	1

**Fig. 5.**
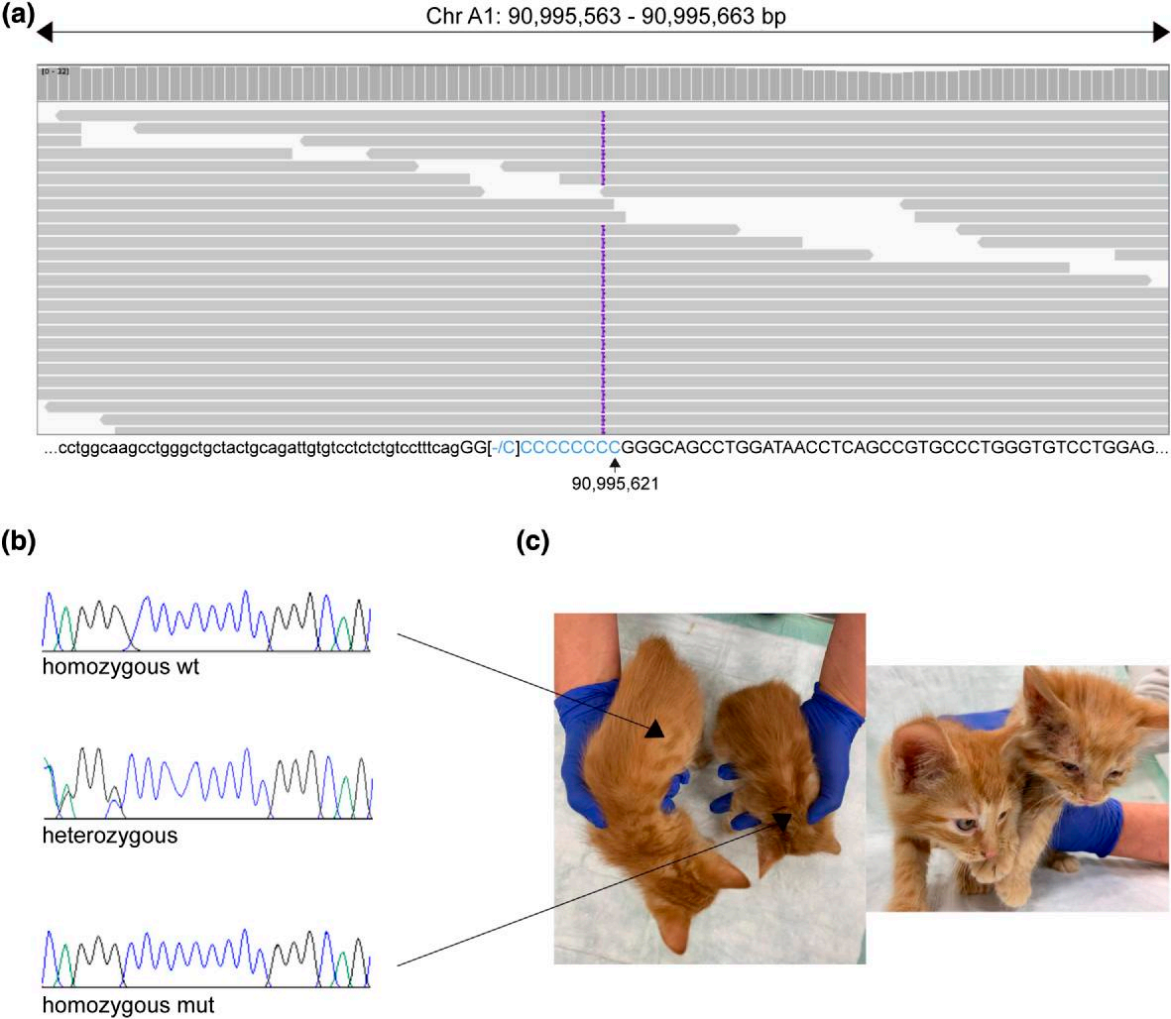
The EDS-associated ADAMTS2 variant on chromosome A1. a) Integrative Genome Viewer (IGV) screenshot of the affected cat's sequence data indicates a one base pair insertion within a poly C stretch. In the IGV alignment, the insertion/duplication is at the left end of this C-stretch. However, according to the 3′-rule of HGVS, the variant is annotated as ChrA1:90,995,621dup. Coordinates refer to the F.catus_Fca126_mat1.0 assembly. Lower case letters indicate intronic, uppercase letters indicate exonic bases. b) Sanger electropherograms of an unaffected (top), a heterozygous (middle), and an affected cat homozygous for the mutant allele (bottom). Please note that Sanger sequencing was conducted using a reverse primer. Therefore, overlapping electropherogram peaks appear to the left of the heterozygous insertion/duplication. c) Phenotype of a healthy kitten (left) and an EDS-affected sibling showing typical skin lesions and growth retardation (right).

Visual inspection of the short-read alignments in IGV (Robinson *et al*. 2011) indicated a homozygous insertion of a single base pair in exon 4 of the 22 annotated exons of the known candidate gene *ADAMTS2* (Fig. 5). This variant can be designated as XM_023254116.2:c.698dup or XP_023109884.2:p.(Ser235Glnfs*4). It truncates nearly 80% of the wild type *ADAMTS2* open reading frame. The genomic variant designation is ChrA1:90,995,621dup (F.catus_Fca126_mat1.0).

**Fig. 6.**
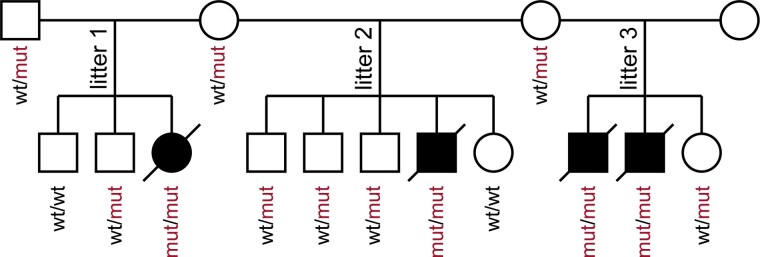
Pedigree of cat family comprising 3 litters with affected kittens, all sired by the same father. Litters 1–3 are consistently numbered in Supplementary Tables 3 and 4. Males are shown as squares and females as circles. Open symbols indicate unaffected cats, which may be heterozygous carriers of the ADAMTS2:c.698dup variant as stated in the individuals’ genotypes. All affected individuals, homozygous for the ADAMTS2 frameshift duplication, are deceased and indicated by filled strikethrough symbols.

The *ADAMTS2* variant was confirmed via PCR and follow-up Sanger sequencing. All available cats (*n* = 31) were genotyped for the variant (Fig. 6; Supplementary Table 3). All 4 affected kittens were homozygous for the mutant allele. Twenty cats were heterozygous, including the parents of affected kittens as well as some of their littermates. The remaining 7 cats were homozygous for the wild type allele. Microsatellite-based parentage testing confirmed the paternity of the suspected tomcat for all 3 litters, in which the 4 affected kittens occurred (Supplementary Table 4).

## Discussion

EDS in humans is known to occur in 13 different subtypes including the autosomal recessive dEDS caused by *ADAMTS2* variants (Malfait *et al*. 2017). So far, in domestic cats only classical EDS (cEDS) caused by autosomal dominant *COL5A1* variants has been characterized at the molecular level (Spycher *et al*. 2018; Kiener *et al*. 2022a).

In this study, we describe a dEDS phenotype in domestic cats due to autosomal recessive loss of function in the *ADAMTS2* gene by a comprehensive clinical, pathological and genetic analysis in a cat family. *ADAMTS2* loss-of-function variants cause autosomal recessive forms of EDS in humans, mice, dogs, cattle, and sheep but have so far not been reported in domestic cats.

During the gross and histological examination of the initial case (first deceased kitten), the skin appeared easily torn. Almost the entire head area and the left side of the neck showed focal alopecia and severe ulcerative purulent dermatitis with serocellular crusts. Similar clinical findings were present in the dermatological examination of the other 3 kittens, with the exception that fresh wounds with less crusting and without secondary pyoderma predominated. In all affected cats, the head, neck and front legs/axilla were most severely affected, which probably resulted from physiological friction and strain to the skin in these body regions. These dermatological findings were consistent with the presence of collagen dysplasia (dermatosparaxia) in other species [overview given by (Vroman *et al*. 2021)]. For example, in hereditary equine regional dermal asthenia, body sites exposed to stress or pressure are most prone to similar lesions (Rashmir-Raven 2013). Comparable dermatological phenotypes can also be observed when caused by variants in ADAMTS2, such as in dogs (Jaffey *et al*. 2022). In previously reported cats with EDS, in which the molecular cause was not identified, skin fragility and predisposition to skin tears was also described as the main clinical finding (Crosaz *et al*. 2013; Hansen *et al*. 2015). Normal handling or even the normal activity of the animal may lead to skin injuries (Counts *et al*. 1980; Crosaz *et al*. 2013; Hargis and Myers 2017).

Hypermobility of the joints, as described for examples in humans and dogs with EDS, has not been described in cats (Mauldin and Peters-Kennedy 2015) similar to the present cases. Histologic examination of the skin showed no abnormalities except for focal loose arrangement of collagen fibers and cleft formation. This might result from the severe ulceration and inflammation and has to be differentiated from subepidermal blistering diseases. Lack of joint hypermobility and variation regarding the caliber of the collagen fibers with irregular, undirected, and loose arrangement have been described as typical for dEDS (Gross *et al*. 2008). Apart from multifocal loose arrangement of collagen fibers, the skin of the necropsied cat showed a regular anatomical morphology; however, histologic findings may vary in cats with collagen dysplasia ranging from no dermal changes up to a thinner dermis with fine collagen fibers separated by an increased amount of ground substance. Normal collagen fibers stain uniformly blue with Masson trichrome stain as in this case, whereas abnormal fibers have segmental red staining areas that are birefringent under polarized light (Butler 1975; Holbrook *et al*. 1980; Sequeira *et al*. 1999; Crosaz *et al*. 2013; Mauldin and Peters-Kennedy 2015).

Due to the postmortem changes in most of the affected kittens, the skin of only one cat was examined by electron microscopy and revealed empty tube appearance of collagen fibers typical for EDS. The inflammatory skin lesions were likely due to secondary infections and not primarily associated with dEDS. The same applies in all likelihood also for the follicular hyperplasia of the mesentric lymph nodes. A hernia diaphragmatica (Fig. 2), also observed in one of the present cases, has been previously described in cats with collagen dysplasia (Benitah *et al*. 2004). It is also possible that the rectal prolapse (Fig. 1b) represented a consequence of the collagen disturbances due to dermatospraxis EDS but might also have resulted from the colo-colic intussusception. The accompanied chronic purulent colitis suggested the presence of a bacterial infection and might have been the cause for intussusception (Uzal *et al*. 2016). Hernia diaphragmatica or rectal prolapse or any other clinical sign except the cutaneous lesions was not present in other kitten affected by EDS.

Different variants within the *ADAMTS2* were already proven to be causative for cases of dermatospraxis EDS in humans (van Damme *et al*. 2016), sheep (Zhou *et al*. 2012; Monteagudo *et al*. 2015; Joller *et al*. 2017), cattle (Colige *et al*. 1999), and dogs (Jaffey *et al*. 2019, 2022). The human ClinVar database lists NM_014244.4(*ADAMTS2*):c.691del as a pathogenic variant. This variant also introduces a frameshift at a position comparable to the feline c.698dup variant. The feline *ADAMTS2* frameshift variant detected herein therefore represents a highly plausible candidate variant for the EDS phenotype in the affected cats. The causality of the *ADAMTS2* frameshift variant is further supported by the perfect cosegregation of genotypes with phenotypes in an extended pedigree with 31 cats, of which 4 were affected.

When we apply the ACMG/AMP consensus criteria for human diagnostics (Richards *et al*. 2015) to the feline *ADAMTS2*:c.698dup frameshift variant, we have one very strong evidence for pathogenicity (null variant in a gene, where loss of function is a known mechanism of disease, PVS1), one moderate criterion (mutant allele is absent from 77 control genomes, PM2), and one supporting evidence (cosegregation with disease in multiple affected members, PP1). Taken together, these 3 lines of evidence allow to classify *ADAMTS2*:c.698dup as pathogenic.

The autosomal recessive disorder analyzed herein phenotypically resembles an EDS form that Hansen *et al*. (2015) already described for a case in Burmese cats (Hansen *et al*. 2015). No molecular genetic analysis was reported in that case. In contrast, different previously identified variants in the *COL5A1* gene were involved in autosomal dominant cEDS cases in cats (Spycher *et al*. 2018; Kiener *et al*. 2022a). Similar to EDS in humans, there are different types of this syndrome in animals that show locus heterogeneity and different modes of inheritance (Malfait *et al*. 2017).

Our analysis suggests that inbreeding within a population of free-roaming farm cats has provoked the outbreak of a lethal recessive disease. The genome of the sequenced case did not show a particularly high level of homozygous variant calls. Nonetheless, the results of our study are in agreement with a more representative study reporting 19% of UK nonpedigree cats with a higher than expected content of homozygous genome regions due to recent inbreeding events (Irving McGrath *et al*. 2021). Potential health risks due to inbreeding should be kept in mind when managing free-roaming cat populations.

## Conclusion

In summary, we describe the *ADAMTS2*:c.698dup frameshift variant as a highly plausible candidate causative variant for dEDS, an autosomal recessive form of EDS in cats. Similar *ADAMTS2* variants have been reported in humans, cattle, sheep, and dogs with dEDS. The functional knowledge from other species and the perfect cosegregation of the genotypes with the phenotype in a medium sized cat family support the causality of the detected *ADAMTS2*:c.698dup variant. Our findings enable genetic testing that can be used to detect healthy carriers and to eradicate this potentially lethal disease from the cat population.

## Supplementary Material

jkad152_Supplementary_Data

## Data Availability

The whole genome sequence data from this study is publicly available from ENA (European Nucleotide Archive). The accessions are listed in Supplementary Table 1. Supplementary Material provided at figshare: https://doi.org/10.25387/g3.22809347. Supplemental material available at G3 online.
